# Characterization of orexin input to dopamine neurons of the ventral tegmental area projecting to the medial prefrontal cortex and shell of nucleus accumbens

**DOI:** 10.1007/s00429-021-02449-8

**Published:** 2022-01-14

**Authors:** Imre Kalló, Azar Omrani, Frank J. Meye, Han de Jong, Zsolt Liposits, Roger A. H. Adan

**Affiliations:** 1 Laboratory of Endocrine Neurobiology, Institute of Experimental Medicine, Eötvös Loránd Research Center, Budapest, 1083 Hungary; 2 Department of Neuroscience, Faculty of Information Technology and Bionics, Pázmány Péter Catholic University, Budapest, 1083 Hungary; 3 Department of Translational Neuroscience, UMC Brain Center, University Medical Center Utrecht, Universiteitsweg 100, 3584 Utrecht, The Netherlands; 4 Department of Neuroscience and Physiology, Sahlgrenska Academy, University of Gothenburg, 40530 Goteborg, Sweden

**Keywords:** Orexin, Dopamine, Ventral tegmental area, Nucleus accumbens, Prefrontal cortex

## Abstract

Orexin neurons are involved in homeostatic regulatory processes, including arousal and feeding, and provide a major input from the hypothalamus to the ventral tegmental area (VTA) of the midbrain. VTA neurons are a central hub processing reward and motivation and target the medial prefrontal cortex (mPFC) and the shell part of nucleus accumbens (NAcs). We investigated whether subpopulations of dopamine (DA) neurons in the VTA projecting either to the mPFC or the medial division of shell part of nucleus accumbens (mNAcs) receive differential input from orexin neurons and whether orexin exerts differential electrophysiological effects upon these cells. VTA neurons projecting to the mPFC or the mNAcs were traced retrogradely by Cav2-Cre virus and identified by expression of yellow fluorescent protein (YFP). Immunocytochemical analysis showed that a higher proportion of all orexin-innervated DA neurons projected to the mNAcs (34.5%) than to the mPFC (5.2%). Of all sampled VTA neurons projecting either to the mPFC or mNAcs, the dopaminergic (68.3 vs. 79.6%) and orexin-innervated DA neurons (68.9 vs. 64.4%) represented the major phenotype. Whole-cell current clamp recordings were obtained from fluorescently labeled neurons in slices during baseline periods and bath application of orexin A. Orexin similarly increased the firing rate of VTA dopamine neurons projecting to mNAcs (1.99 ± 0.61 Hz to 2.53 ± 0.72 Hz) and mPFC (0.40 ± 0.22 Hz to 1.45 ± 0.56 Hz). Thus, the hypothalamic orexin system targets mNAcs and to a lesser extent mPFC-projecting dopaminergic neurons of the VTA and exerts facilitatory effects on both clusters of dopamine neurons.

## Introduction

Loss of orexin (also known as hypocretin) (de Lecea et al. 1998; Sakurai et al. 1998) signaling results in narcolepsy and increased orexin signaling has been implicated in arousal, feeding and addiction (Horvath and Gao 2005; Baimel and Borgland 2017; James et al. 2017). Orexin neurons reside in the lateral hypothalamic region and project throughout the brain (Peyron et al. 1998), including to the ventral tegmental area (VTA). VTA neurons are implicated in reward processing and motivation and they target structures such as the medial prefrontal cortex (mPFC) and nucleus accumbens (NAc) (Tzschentke and Schmidt 2000). Orexins, via orexin 1 and 2 receptors (Trivedi et al. 1998; Sakurai et al. 1998), excite VTA dopamine neurons (Korotkova et al. 2003; Vittoz et al. 2008). VTA neurons are diverse regarding their inputs, electrophysiological properties, and projection specificity (Swanson 1982; Albanese and Minciacchi 1983; Lammel et al. 2012; Yang et al. 2018; Farassat et al. 2019). Orexin increases the firing rate of VTA dopamine neurons projecting the NAc, but not to the basolateral amygdala (Baimel et al. 2017). VTA dopamine neurons projecting to the NAc and to the mPFC are activated by orexin and implicated in the arousal (España et al. 2001; Vittoz and Berridge 2006; Vittoz et al. 2008). However, it has not been explored whether the orexin innervation of VTA dopamine neurons projecting to the mPFC differs from those projecting to the shell part of the NAc (NAcs).

Although orexin-containing axons are present throughout the VTA (Fadel and Deutch 2002), only 15% of them were found to make appositional contacts in the VTA and even less (5%) to form definitive synaptic specializations (Balcita-Pedicino and Sesack 2007). Because the majority of orexin immunoreactive (IR) axons does not establish synaptic connections, it is conceivable that orexin release in this region is largely extra-synaptic (Balcita-Pedicino and Sesack 2007).

Here, we investigated whether subpopulations of dopaminergic VTA neurons projecting either to the mPFC or the mNAcs receive differential input from orexin neurons and whether orexin has differential electrophysiological effects on these DA neuron populations.

## Materials and methods

### Animals

Wistar rats (Charles River, Germany, *n* = 15) weighing 200–225 g were used for immunohistological quantifications and Pitx3-EGFP heterozygous mice (P80–P90) were used for electrophysiology experiments (as electrophysiological recordings from dopamine neurons in the VTA is more feasible in mice than in rats, as we could visualize dopamine neurons by their fluorescence in Pitx3-EGFP mice). Animals were housed in a temperature (21 ± 2 °C) and humidity (60–70%) controlled room under a 12 h reversed light/dark cycle (lights on 7.00 h). They had ad libitum access to normal chow and drinking water. All experiments were performed in accordance with Dutch laws (Wet op de Dierproeven, 1996) and European regulations (Guideline 86/609/EEC), and were approved by the Animal Ethics Committee of Utrecht University and the Animal Welfare Committee of Institute of Experimental Medicine (Permission No. A5769-01).

### Stereotaxic surgeries

**Fig. 1:**
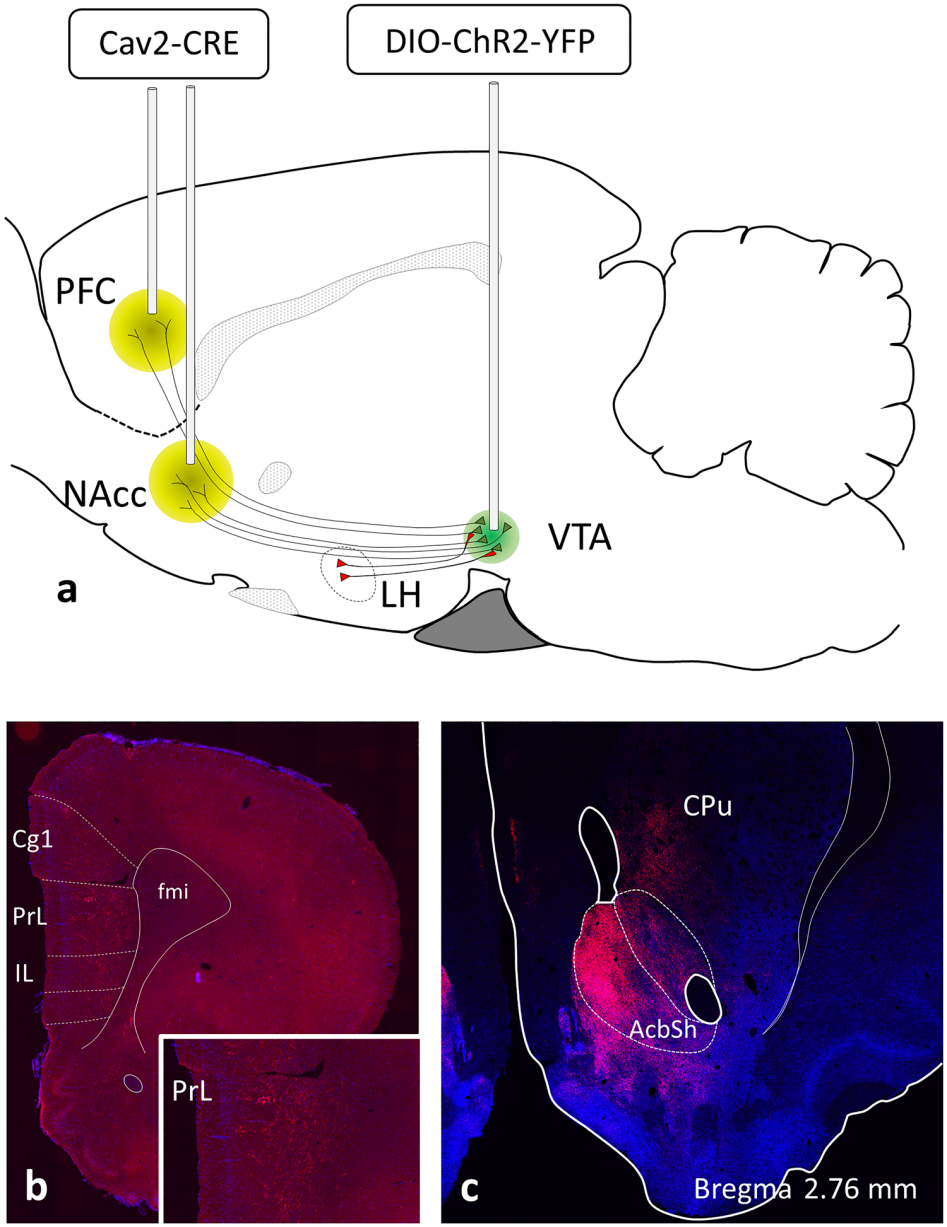
Illustration of virus-based tracing of VTA neurons projecting to the medial prefrontal cortex or shell of nucleus accumbens. **a** Position of injection needles to deliver the retrograde tracer canine adenovirus (Cav2-CRE) and the adeno-associated virus (DIO-ChR2-YFP). **b**, **c** The projecting axons of ChR2-YFP-expressing neurons of the VTA appear in the prefrontal cortical areas and the shell part of nucleus accumbens marking the injection sites for Cav2. The inset is a higher power image of the prelimbic area of the PFC demonstrating the axon distribution of projecting neurons. *Cg1* cingulate cortex, region 1, *CPu* caudate-putamen, *fmi* forceps minor, *IL* infralimbic area, *LH* lateral hypothalamic area, *NAcs* nucleus accumbens, shell part, *mPFC* medial prefrontal cortex, *PrL* prelimbic area, *VTA* ventral tegmental area

All experimental animals were anaesthetized with ketamine (75 mg/kg) and medetomidine (1 mg/kg). Lidocaine (100 mg/ml, AstraZeneca BV, the Netherlands) was sprayed on the skull to provide local anesthesia. They were then placed in a stereotaxic frame (David Kopf Instruments, United State) and a craniotomy was carried out. Then, viral injections were performed using a 2 µl Hamilton syringe controlled by an injection pump at a rate of 0.1 µl/min. For quantitative immunohistology, 10 rats were bilaterally injected with 1 μl of AAV5-DIO-hChR2(H134R)-eYFP (1 × 10^12^ genomic copies/ml; UNC Vector Core, USA) in the VTA (AP − 5.4 mm, ML 2.2 mm, DV − 8.9 mm from bregma, angle 10°) and 1 μl of retrograde Cav2-Cre virus (1.8 × 10^12^ genomic copies/ml; IGMM, France) targeting the medial division of the shell part of nucleus accumbens (mNAcs) (AP 1.2 mm, ML 2.8 mm, DV − 7.5 mm from bregma, angle 10°) or mPFC (AP 2.7 mm, ML 1.4 mm, DV − 4.9 mm from bregma, angle 10°; (Paxinos and Watson 2005), see Fig. 1 for a schematic illustration of the used approach). For electrophysiological recordings, to target NAcs-projecting DA neurons four male mice were bilaterally injected with 300 nl of AAV-DIO-mCherry (1 × 10^12^ genomic copies/ml; UNC Vector Core, USA) in the VTA (AP − 3.2 mm, ML 1.5 mm, DV − 4.8 mm from bregma, angle 15°) and 300 nl of retrograde Cav2-Cre virus in the mNAcs (1.8 × 10^12^ genomic copies/ml; AP 1.4 mm, ML 1.9 mm, DV − 4.2 mm from bregma, angle 5°) (Paxinos and Franklin 2013). To target mPFC-projecting DA neurons five male mice were bilaterally injected with 200 nl of retrogradely transported herpes simplex virus (HSV) expressing mCherry (4 × 10^8^ genomic copies/ml; Dr. R. Nee, McGovern Institute) supplemented with 20 × diluted latex red RetroBeads (Lumafluor) to mark injection sites, into the mPFC (double injection at two different sites; AP 1.95 mm, ML 0.60 mm, DV − 1.85 and AP 2.25 mm, ML 0.70 mm, DV − 2.35 mm from bregma, angle 15°) (Paxinos and Franklin 2013). Injection needles were left in place for 10 min to prevent backflow. The skin was then sutured, the animals received carprofen (5 mg/kg, s.c., Carporal, AST Farma BV, the Netherlands) and were kept on a heating pad until they recovered from anesthesia. Animals were allowed to recover for at least three weeks before experiments were performed.

### Histology and confocal microscopy

#### Tissue preparation for detecting the viral fluorescent tracer and immunofluorescence

The animals were perfused transcardially with phosphate-buffered saline (PBS; 0.1 M) containing 4% paraformaldehyde (PFA). The brains were removed, post-fixed for 2 days, and transferred into 30% sucrose for cryoprotection, then, 30 µm thick coronal sections were cut on a freezing microtome by collecting every sixth section into the same well.

### Evaluation of viral tracing of projecting axons

A group of sections was mounted from each brain with mPFC (*n* = 5) or mNAcs (*n* = 4) injections of the Cav2-Cre viral tracer. Brains showing YFP-labeled processes concentrated in the mPFC or the mNAcs at the injection sites (Fig. 1b and c) indicated successful recombination of the floxed channel rhodopsin-YFP encoding sequences in VTA neurons, and were selected for subsequent multiple-label immunofluorescence staining for YFP, TH and orexin B.

### Triple-label immunofluorescence

After the endogenous peroxidase activity had been quenched with 0.5% hydrogen peroxide (20 min), sections were permeabilized with 0.5% Triton X-100 (23,472-9, Sigma, 20 min), and treated with 2% normal horse serum (20 min) to reduce non-specific antibody binding. All treatments and interim rinses in PBS (3 × 5 min) were carried out at room temperature, except for incubation in the primary antibodies and fluorochromes, which was carried out at 4 ℃. Sections were incubated in a cocktail of the primary antibodies for 72 h and the fluorochrome-labeled secondary antibodies overnight with a two hour-rinse in tris(hydroxymethyl)aminomethane (TRIS) in between. To maximize the visualization of cellular borders of mPFC- or mNAcs-projecting neurons, the membrane-localized YFP signal was enhanced by its immunofluorescent detection applying either CY3 or FITC-labeled secondary antibodies. For immunohistochemical triple labeling, rabbit anti-GFP (#AB10145, RRID:AB_1587096, Millipore, 1:5000), mouse anti-TH (#22941, RRID:AB_572268, Immunostar; 1.6000) and goat anti-orexin B (sc-8071; C-19; RRID:AB_653612, Santa Cruz Biotech Inc., 1∶50,000) primary antibodies, and FITC-donkey-anti-rabbit IgG (H + L) (#711-095-152, RRID:AB_2315776, Jackson ImmunoResearch Laboratories, 1: 500), CY5-donkey-anti-mouse IgG (#715-175-151, RRID:AB_2340820, Jackson ImmunoResearch Laboratories, 1:2000) and CY3-donkey-anti-goat IgG (H + L), 705-165-147, RRID:AB_2307351, Jackson ImmunoResearch Laboratories, 1:3000) secondary antibodies were used to stain sections containing the VTA. Sections were then rinsed in TRIS (2 h), mounted onto glass slides and cover slipped with Moviol.

### Immunohistochemical controls

The orexin antiserum has been thoroughly characterized in rat brain sections (Deurveilher et al. 2006; Bullmann et al. 2010). The staining pattern provided by the TH antiserum agreed with the staining pattern resulted in using other TH antisera (Stott et al. 2013) and the known distribution of DA neurons in the pars compacta of the SN and in other brain regions including the VTA (Bubar and Cunningham 2007). The staining pattern generated by the GFP antiserum perfectly matched the distribution of GFP positive perikarya and processes. The previously reported specificity tests of these primary antisera have been supplemented in the current experiment with a negative control test to optimize the working dilutions; this was carried out by increasing the dilutions of the primary antisera, which resulted in a commensurate decrease and eventual disappearance of the immunostaining. Secondary antibodies were designed for multiple labeling and pre-absorbed by the manufacturer with immunoglobulins from several species, including those in which the other primary antibodies had been raised.

### Confocal laser microscopy

**Fig. 2:**
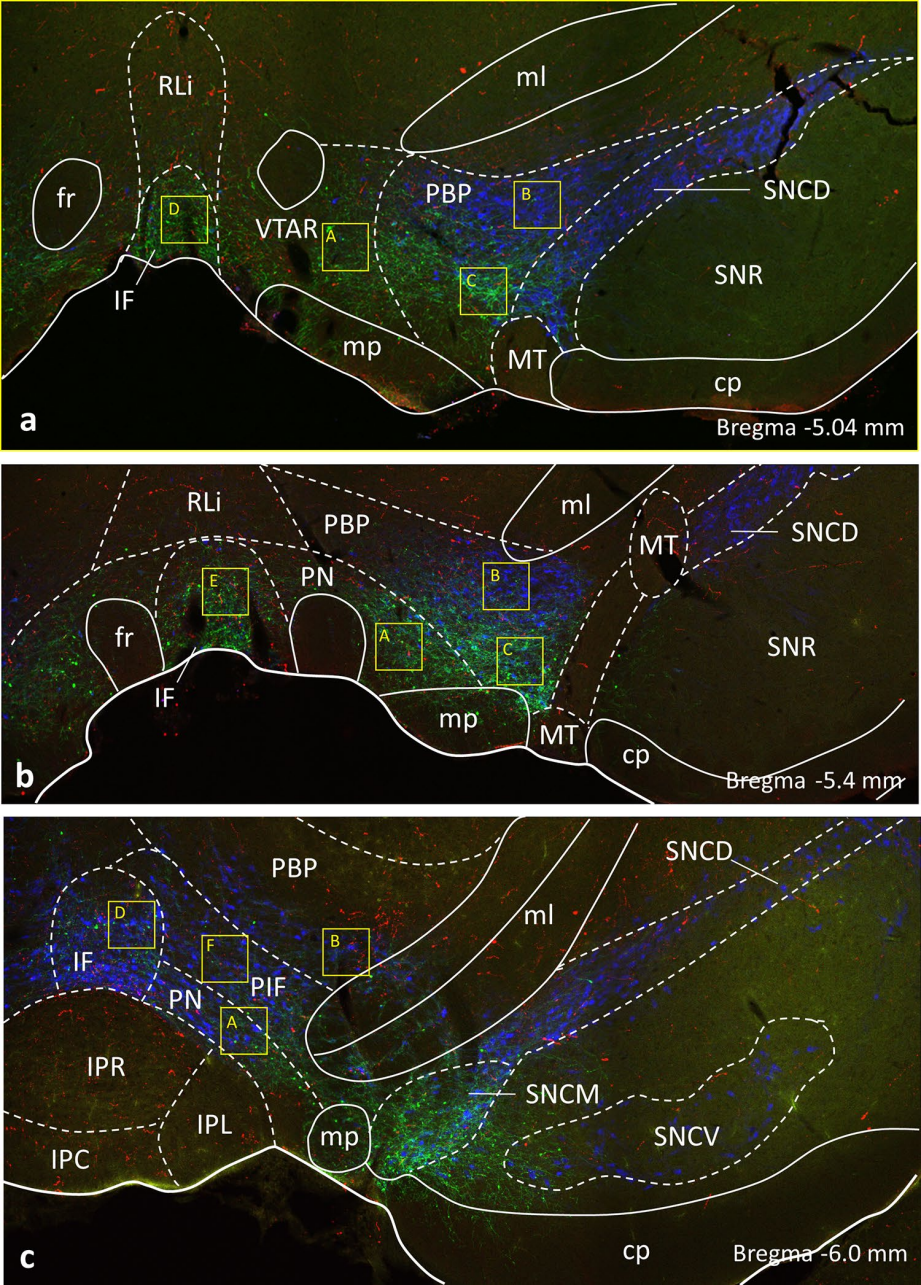
Mosaic confocal images of rat brain sections labeled immunohistochemically for yellow fluorescent protein (YFP; in green), tyrosine hydroxylase (TH; in blue) and orexin B (in red) demonstrating the major subdivisions of the ventral tegmental area (VTA) i.e., rostral VTA (VTAR); parabrachial pigmented (PBP) subdivisions; interfascicular (IF); paranigral (PN); parainterfascicular (PIF) nuclei at three rostro-caudal levels (**a–c**) after viral targeting the mNAcs-projecting DA neurons. Boundaries of brain regions (solid and dashed lines) correspond to those delineated in the rat brain atlas (Paxinos Rat Brain Atlas, 5th Edition). Regions of quantitative analyses for detecting orexin B-IR appositions on labeled VTA neurons (A-F) are marked by yellow boxes (X–Y dimensions correspond to the maximum field scanned by the laser at 60 × magnification

**Fig. 3:**
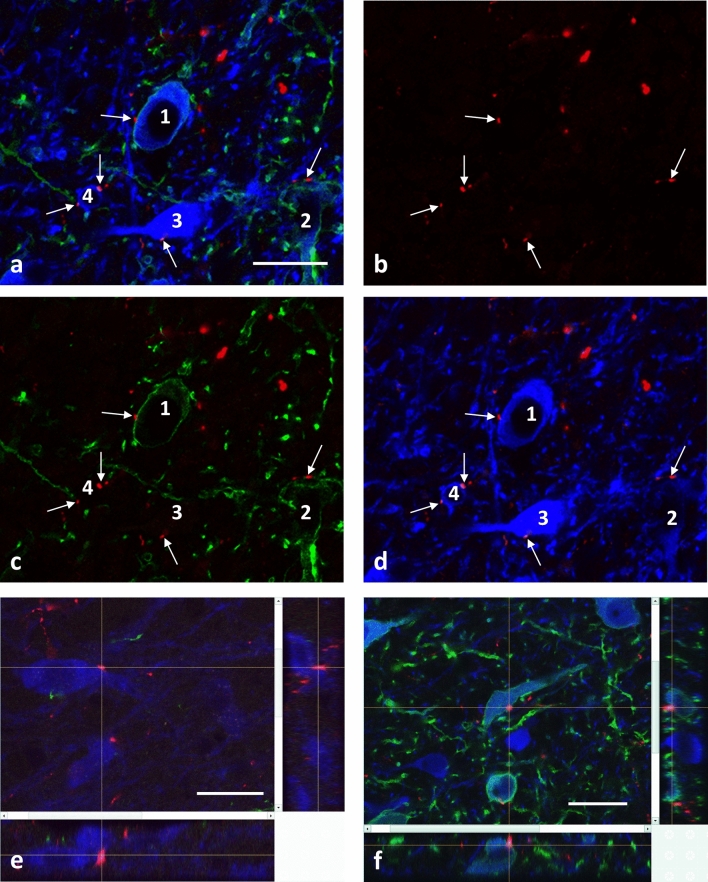
Confocal microscopic images of the parabrachial pigmented subdivision (PBP) of the VTA, immunohistochemically labeled for yellow fluorescent protein (YFP; in green), tyrosine hydroxylase (TH; in blue) and orexin B (in red). YFP is expressed in certain VTA neurons due to their local infection by AAV-DIO-ChR2-YFP, and distant infection of their axon terminals in the medial shell part of nucleus accumbens (**a**–**d**) or the medial prefrontal cortex (**f**) by the retrogradely transported Cav2-CRE. Four TH-IR perikarya, and several TH-IR neural processes are visible in **a**, some of which belong to neurons projecting to the nucleus accumbens, as recognized by the green channel rhodopsin-YFP appearing in the cell membrane (**a**, **c**; cells 1 and 2). Orexin B-IR axon varicosities are present among these neurons (**a–d**), and form appositions on TH-IR neurons (arrows) projecting to the nucleus accumbens (1 and 2) or other brain regions (3 and 4). **e–f** Projection images of orexin B-IR axon varicosities (arrows) on proximal dendrite of a TH-IR neuron (**e**) or a mPFC-projecting neuron TH-IR neuron (**f**). Scale bar: 25 µm

Triple-labeled sections at three rostro-caudal levels (Fig. 2a–c; Bregma 5.04; − 5.40 and − 6.00, respectively) were selected from each brain and scanned using a NIKON A1 confocal microscope. Multiple stacks of optical slices (1024 × 1024 pixels, z-steps 0.6 μm) were obtained using a Plan Apochromat 63x/1.4 NA oil immersion objective. Scans (1–5) were generated from the major subdivisions of the ventral tegmental area (VTA) i.e., rostral VTA (VTAR); parabrachial pigmented (PBP) subdivisions; interfascicular (IF); paranigral (PN); and parainterfascicular (PIF) nuclei (Paxinos and Watson 2005). To find the boundaries of the different subnuclei of VTA, the size and shape of the TH-IR neuronal groups were examined and the position of other landmarks (i.e., myelinated axonal bundles, tracts) was referenced to the rat brain atlas. Regions of interest (ROI; 50,589 μm^2^) containing TH-YFP, TH- and YFP cells in the rostral VTA (VTAR; *n* = 1); parabrachial pigmented (PBP; *n* = 5) subdivisions; interfascicular (IF; *n* = 3); paranigral (PN; *n* = 2); parainterfascicular (PIF; *n* = 1) nuclei were scanned (to a depth of 19–20 μm) in one side of the selected three sections of the VTA regions. The scans were analyzed for the number of orexin B-IR fiber varicosities in appositions to the perikarya and the proximal dendrites of three basic cell types i.e., those, which were immunoreactive for TH and projected either to mPFC or mNAcs (TH-YFP-cells), those, which were immunoreactive for TH, but were not targeted by the retrogradely transported CAV-CRE viruses (TH cells), and finally those, which were TH-immunonegative, but were targeted by the retrogradely transported CAV-CRE viruses (YFP cells). The sequentially recorded green, red and far-red channels were merged and displayed with the NIS Elements. The number of TH-YFP, TH**-** and YFP cells contacted by orexin B-IR processes and the incidence of orexin B-IR appositions on TH-YFP, TH- and YFP cells were quantified. Each perikaryon showing TH- and/or YFP-immunoreactivities and receiving orexin B-IR afferent(s) has been marked. Axon appositions (defined by the absence of any visible gap between the juxtaposed profiles verified in orthogonal views, e.g., Fig. 3e–f) and immunoreactive perikarya were numbered. Both perikaryal and dendritic appositions were counted; dendrites were considered only if their connections to the perikarya were traceable. To avoid double counting of perikarya or axon appositions, immunoreactive profiles appearing repeatedly in the overlapping parts of neighboring Z-stacks or neighboring optical slices of the Z-stacks were identified and encoded with the same profile number. Data are presented as mean ± SEM. Group differences were assessed by *t* test. Statistical significance was set at *p* < 0.05.

### Electrophysiology

Horizontal slices of the midbrain (250 µm) were prepared from Pitx3-EGFP mice, using a vibratome (Leica VT1200S, Leica Microsystems) in ice-cold modified artificial cerebrospinal fluid (ACSF) containing (in mM): 92 N-methyl-D-glucamine (NMDG), 2.5 KCl, 1.25 NaH_2_PO_4_, 30 NaHCO_3_, 20 HEPES, 25 glucose, 2 thiourea, 5 Na-ascorbate, 3 Na-pyruvate, 0.5 CaCl_2_.4H_2_O, and 10 MgSO_4_.7H_2_O, bubbled with 95% O_2_ and 5% CO_2_ (pH 7.3–7.4). Slices were initially recovered in carbogenated modified ACSF for 15 min at 34 °C and then, transferred into a holding chamber containing standard ACSF (in mM): 126 NaCl, 3 KCl, 2 MgSO_4_, 2 CaCl_2_, 10 glucose, 1.25 NaH_2_PO_4_ and 26 NaHCO_3_ bubbled with 95% O_2_ and 5% CO_2_ (pH 7.3) at room temperature for at least 1 h. The slices were transferred one at a time to the recording chamber perfused with standard ACSF continuously bubbled with 95% O_2_ and 5% CO_2_ at 30–32 °C. Whole-cell patch-clamp recordings were made from VTA dopamine neurons visualized with an Olympus BX51W1 microscope using infrared video microscopy and differential interference contrast (DIC) optics, using a Rolera XR camera (QImaging). VTA dopaminergic neurons were identified by green fluorescence, and VTA neurons projecting to either mNAcs or mPFC were recognized by red fluorescence. Recordings were made from VTA neurons expressing both green and red fluorescence.

Patch electrodes were pulled with a Sutter P-97 from borosilicate glass capillaries (Harvard Apparatus, 1.5 mm OD), with a resistance of 3–5 MΩ when filled with intracellular solutions. Internal solution contained (in mM): 140 K-gluconate, 1 KCl, 10 HEPES, 0.5 EGTA, 4 MgATP, 0.4 Na_2_GTP, 4 phosphocreatine (pH 7.3 with KOH). Signals were amplified, filtered at 3 kHz, and digitized at 10 kHz using EPC-9 and EPC-10 patch-clamp amplifiers and PatchMaster v2 × 73 (HEKA Elektronik) software. No series resistance compensation was used. Resting membrane potential was measured in zero current mode (*I* = 0) immediately after obtaining whole-cell access. The basic electrophysiological properties of the cells were determined from the voltage responses to a series of hyperpolarizing and depolarizing square current pulses. To determine the direct postsynaptic effect of orexin A on VTA dopamine neurons, synaptic inputs onto these neurons were blocked using inhibitors of AMPA/Kainate (CNQX 10 µM; Tocris, Bristol, UK), NMDA (APV 50 µM; Tocris, Bristol, UK) and GABA_A_ receptors (bicuculline 20 µM; Tocris, Bristol, UK). Orexin-A (Tocris, Bristol, UK) was dissolved in ACSF at a concentration of 100 nM and was applied to the bath through perfusion. Based upon similar experiments in literature, we chose 100 nM as a concentration for these experiments as this is a saturating concentration of orexin used before in similar experiments (Baimel et al. 2017). After 10 min baseline recording, orexin was applied for at least 5 min and firing frequency was determined during this period and contrasted with baseline. In the case of VTA dopamine neurons projecting to the mPFC which could be quiescent during baseline, these recordings were done in the presence of a constant depolarizing injected current to ensure action potential firing already during baseline period, allowing for modulation of orexin application. VTA dopamine neurons projecting to mNAcs were recorded with zero current mode, as they were not quiescent. Passive and active membrane properties were analyzed with Igor Pro 7 (Wavemetrics) software. Effects of orexin on firing frequency and membrane properties were assessed using Repeated Measures ANOVA in SPSSv27 (IBM).

## Results

### Characterization of mPFC- and mNAcs-projecting dopaminergic neurons in the VTA

**Fig. 4:**
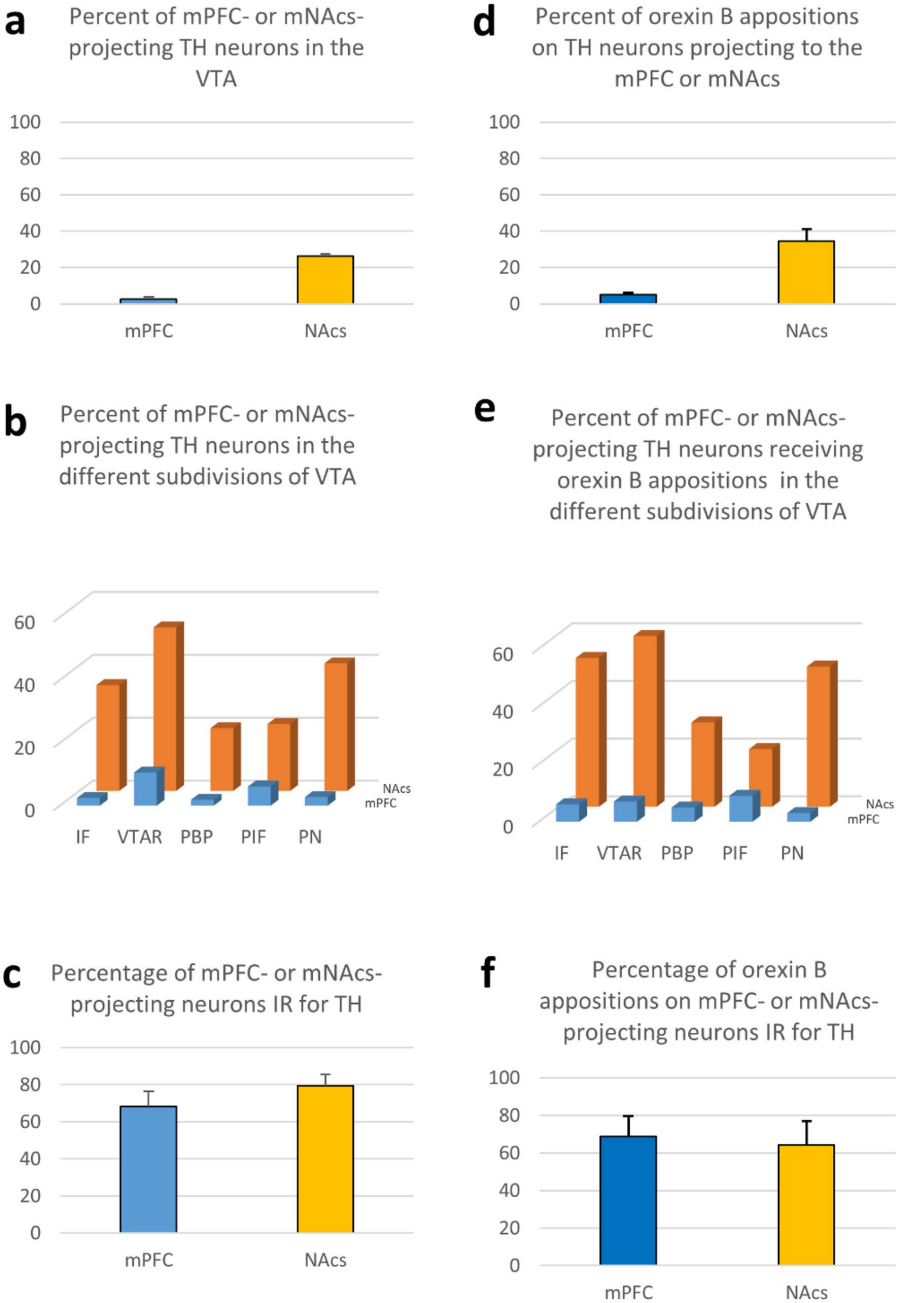
Graphical demonstration of quantitative data obtained by the analyses of 99 Z-series of optical slices from the VTA region of 9 rat brains, within the mNAcs- and mPFC-projecting neurons were traced and characterized for orexin B input. **a** Percentage of TH neurons projecting to the NAcs or mPFC. **b** Percentage of mPFC- or NAcs-projecting TH neurons in the different subdivisions of VTA. **c** Percentage of NAcs- or mPFC-projecting neurons immunoreactive for TH. **d** Percentage of TH neurons receiving orexin B appositions and projecting to the NAcs or mPFC. **e** Percent of mPFC- or NAcs-projecting TH neurons receiving orexin B appositions in the different subdivisions of VTA. **f** Percentage of NAcs- or mPFC-projecting neurons receiving orexin B appositions and immunoreactive for TH

To visualize VTA neurons projecting to the mPFC and/or to mNAcs, a dual vector strategy was used (Boender et al. 2014). Thus, Cav2-Cre was injected into the projection target sites and a cre-dependent AAV-expressing channelrhodopsin fused to eYFP (AAV5-DIO-hChR2(H134R)-eYFP) into the VTA (Fig. 1). Immunohistochemical triple-labeling for the (YFP), tyrosine hydroxylase (TH) and orexin B revealed TH-IR and/or YFP-IR neurons and orexin B-IR axon varicosities in all major subdivisions of the ventral tegmental area (VTA) i.e., rostral VTA (VTAR) and parabrachial pigmented (PBP) subdivisions, interfascicular (IF), paranigral (PN) and parainterfascicular (PIF) nuclei (Fig. 2). The prevalence of the retrogradely traced neurons (YFP-positive) varied in the different subdivisions. The highest number of them appeared in the PBP, but they were substantially present also in the IF by both the mNAcs and mPFC injections. Retrogradely-traced cells were also distributed in the caudal VTA exhibiting a relatively high prevalence in the PN subdivision by the mNAcs injections, and in the PIF subdivision by the mPFC injections, respectively. The confocal microscopic analyses of the selected ROIs in the different subdivisions of the VTA revealed proportionally different TH-positive, dopamine neurons to express YFP (Fig. 4b). Quantitative analyses of the TH- and/or YFP-IR neurons revealed, that 26.4 ± 3.8% of all counted TH-positive, DA neurons were traced by the retrogradely transported virus from the mNAcs, and 2.7 ± 0.6% of them were targeted from the mPFC (Fig. 4a). A large fraction, but not all mPFC- (68.3 ± 8.2%) and mNAcs- (79.6 ± 5.8%) projecting neurons were TH-immunopositive (Fig. 4c).

### Characterization of orexin B neuronal input to dopaminergic VTA neurons

Orexin B-IR axon varicosities were found in juxtaposition to TH-IR perikarya and dendrites (Fig. 3). Appositions were observed on mPFC- (Fig. 3f), as well as mNAcs- (Fig. 3a–d) projecting TH-immunopositive neurons (TH-YFP neurons), on single labeled TH-IR (TH neurons) or YFP-positive, mPFC- or NAcs-projecting, TH-immunonegative neurons. The prevalence of the orexin B appositions on the retrogradely-traced neurons (YFP-positive) varied in the different subdivisions. The highest number of them appeared in the PBP, but they were substantially present also in the IF by both the mNAcs and mPFC injections. Orexin B appositions on the retrogradely-traced cells were also in the caudal VTA exhibiting a relatively high prevalence in the PN subdivision by the mNAcs injections, and in the PIF subdivision by the mPFC injections, respectively. Quantitative analyses of orexin B-IR appositions on mPFC- or mNAcs-projecting dopaminergic neurons in the sampled regions of the VTA revealed that the mNAcs-projecting, TH-positive perikarya received about one third of all orexin B-IR appositions (34.5 ± 6.7%), whereas the mPFC-projecting TH-positive perikarya received only a small fraction of all orexin B-IR appositions (5.2 ± 1%) (Fig. 4d). The contribution of the dopaminergic subpopulation of VTA, receiving orexin B input and projecting to the mPFC-, or NAcs varied within the different subdivisions (Fig. 4e). Out of all mPFC- or mNAcs-projecting neurons which received orexin B-IR appositions, respectively, 68.9 ± 10.8 or 64.4 ± 12.6% were also immunoreactive for TH **(**Fig. 4f**)**. The number of orexin B-IR fiber varicosities found on the perikarya and the proximal dendrites of mPFC- or mNAcs-projecting neurons did not differ significantly from each other (on somata 53.1 ± 16.9 vs. 57.2 ± 3.4% and on dendrites 46.8 ± 16.9 vs. 42.8 ± 3.4%, respectively). The density of orexin innervation was similar for the mPFC- and mNAcs-projecting neurons (mPFC 2.2 ± 0.7, mNAcs 1.8 ± 0.3).

### Effect of orexin A on VTA dopamine neurons projecting to mNAcs or mPFC

**Fig. 5:**
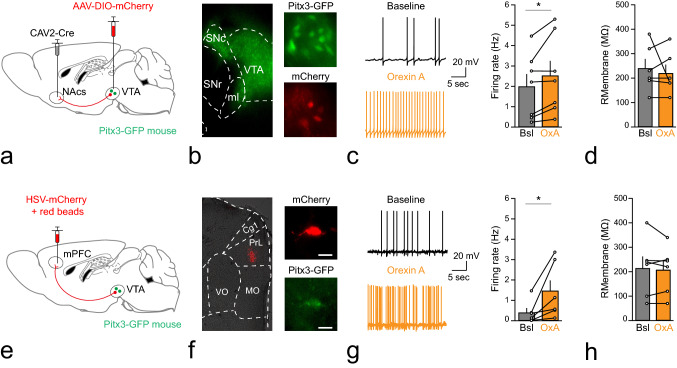
Acute effects of orexin A on VTA dopamine neurons projecting to either mPFC or mNAcs. **a** Schematic representation of viral strategy to target VTA dopamine neurons projecting to mNAcs. **b** Representative histology examples of Pitx3-GFP mice showing GFP in the VTA and substantia nigra, pars compacta (SNc) (left), containing green fluorescent protein-expressing dopamine neurons in VTA (top right) and mNAcs-projecting cells (bottom right). **c** Representative traces of current clamp recordings from VTA dopaminergic neurons retrogradely-labeled from the mPFC, during baseline conditions (black, top) and after washing in of 100 nM of orexin A (orange, bottom). Bar graph quantification, with individual cells shown as circles, of the effect of orexin A on the population of mNAcs-projecting VTA dopamine cells. **d** Bar graph quantification, with individual cells shown as circles, of the effect of orexin A on the membrane resistance of mNAcs-projecting VTA dopamine neurons. **e** Schematic representation of viral strategy to target VTA dopamine neurons projecting to mPFC. **f** Representative histology examples of injection site of HSV-mCherry (with 20 × PBS-diluted latex red RetroBeads) in the prelimbic (PrL) area of the mPFC (left). Example of mCherry expression in a VTA neuron in a Pitx3-GFP mouse (top right) and expression of GFP in the same neuron (bottom right). White scale bars indicate 10 µm. **g** as (C) but for mPFC-projecting VTA dopamine neurons. **h **As (D) but for mPFC-projecting VTA dopamine neurons. *p < 0.05

We next addressed whether orexin would differentially affect firing levels of VTA dopamine neurons projecting to either the shell of nucleus accumbens or mPFC. We used Pitx3-GFP mice with expression of EGFP in midbrain dopamine neurons (Zhao et al. 2004; Labouebe et al. 2007; Maxwell et al. 2005) which we injected with retrograde viral vectors. In the first series of experiments, we combined Cav2-Cre injections targeted to the mNAcs with a VTA-targeted AAV driving cre-dependent mCherry expression (Fig. 5a, b). We prepared brain slices of these mice and performed whole-cell current clamp recordings from fluorescently double-labeled VTA dopamine neurons (i.e., red fluorescence reflecting VTA neurons projecting to the NAc, and green fluorescence reflecting VTA dopamine neurons). These neurons exhibited capacitance values of 46 ± 5.48 pF (Ncells = 6, Nmice = 4). Recordings were performed in the presence of ionotropic glutamate and GABA receptor blockers (CNQX 10 µM, APV 50 µM and bicuculline 20 µM). After establishing a baseline firing frequency, we bath applied orexin-A (100 nM) and evaluated consequences for firing rate in the first five minutes. Orexin-A significantly increased the firing frequency of these neurons from 1.99 ± 0.61 Hz to 2.53 ± 0.72 Hz (Fig. 5c; F(1,6) = 6.14, *p* < 0.05; Repeated Measures; Ncells = 7, Nmice = 4), without affecting the membrane resistance (Fig. 5d; F(1,5) = 0.287, *p* = 0.62; Repeated Measures; Ncells = 6, Nmice = 4).

We next evaluated the effect of orexin A on VTA dopamine neurons projecting to the mPFC. We labeled these neurons using an mPFC-targeted retrogradely traveling HSV driving mCherry (Bartonjo and Lundy 2020; Neve 2012), (supplemented with diluted red latex beads to mark the injection site (Fig. 5e, f). These VTA dopamine neurons projecting to mPFC exhibited capacitance values (19.15 ± 2.81 pF; Ncells = 6, Nmice = 4) that were lower than that of the NAc-projecting VTA dopamine neurons [F(1,10) = 19.03; *p* < 0.001; One-Way ANOVA], suggesting a smaller cell size. Again, after establishing a baseline firing frequency, we bath applied orexin-A (100 nM) and evaluated consequences for firing rate. Also, in these neurons orexin-A significantly increased the firing frequency, from 0.40 ± 0.22 Hz to 1.45 ± 0.56 Hz [Fig. 5g; F(1,5) = 6.786, *p* < 0.05; Repeated Measures; Ncells = 6, Nmice = 5], without affecting the membrane resistance [Fig. 5h; F(1,5) = 0.326, *p* = 0.59; Repeated Measures; Ncells = 6, Nmice = 5].

The effect of orexin A on firing frequency on VTA dopamine neurons did not differ based on projection target [Repeated Measures orexin-A × Projection interaction F(1,11) = 1.363, *p* = 0.27; Repeated Measures Main effect orexin A F(1,11) = 13.08, *p* < 0.01]. Overall, this suggests that orexin input can increase activity of VTA dopamine neurons projecting either to mNAcs or to mPFC.

## Discussion

Orexins have been implicated in physiological and behavioral processes associated with high-arousal environmental conditions (Vittoz and Berridge 2006). They accomplish these in part via interactions with ascending modulatory systems originating from the VTA, including the A10 dopaminergic cell group *(*Vittoz et al. 2008*)*. The regulatory centers of reward processing are modulated by orexin-synthesizing neurons distributed in the lateral hypothalamus. In this study, we analyzed the regulatory role of orexin upon dopaminergic neurons of the VTA projecting to the mPFC and mNAcs in rodents. Although neuroanatomical studies showed a high degree of co-expression of TH and the dopamine transporter (DAT) in the midbrain dopaminergic neurons (A8-10), this does not exclude the possibility for other neurotransmitters (such as glutamate) to convey signal towards the mPFC and nACs in response to orexin input. We found that (1) A higher percentage of dopaminergic neurons of the VTA is wired to the medial division of the shell part of the nucleus accumbens than to the neuronal circuits of the medial prefrontal cortex, and consequently, a higher proportion of orexin axons in the VTA is engaged to the innervation of mNAcs-projecting dopamine neurons than those providing dopamine supply for the medial prefrontal cortex. (2) The dopaminergic axonal outflows from the VTA to these distinct forebrain loci represent a high percentage of all projecting neurons and most of these dopaminergic neurons controlling the mPFC and the mNAcs are innervated by orexin axons. (3) The proportion of TH neurons and those TH neurons which receive orexin B input in the full population of mPFC- or NAcs -projecting TH neurons is almost identical and finally, (4) The firing rate of mPFC- and mNAcs-projecting DA neurons of mouse VTA is similarly increased by bath application of orexin-A in acute brain slice preparations. It was demonstrated earlier that orexin’s effect on VTA DA neurons was blocked by administration of the orexin-1 antagonist SB 334,867, confirming the involvement of orexin-1 receptors in mediating this effect (Baimel et al. 2017).

### Orexin innervation targets dopaminergic neurons projecting from the VTA to the medial prefrontal cortex

The lateral hypothalamus is rich in orexin-synthesizing neurons (Peyron et al. 1998). From a single precursor protein two isoforms are formed by cleavage: orexin A and orexin B (de Lecea et al. 1998; Sakurai et al. 1998). They act on G-protein coupled receptors (OX1 and OX2) (Sakurai et al. 1998; Trivedi et al. 1998) and influence basic neuronal mechanisms regulating wakefulness, food intake, reward processing, addiction, locomotion and mood (Horvath and Gao 2005; Aston-Jones et al. 2009; Smith et al. 2010; Baimel and Borgland 2017; James et al. 2017). The orexin axon outflow reaches many parts of the neuroaxis including the ventral tegmental area, the nucleus accumbens and the prefrontal cortex, the principal components of the reward system (Tzschentke and Schmidt 2000). Orexin terminals are also capable of releasing co-modulators, the neuropeptide dynorphin (Chou et al. 2001) and the excitatory neurotransmitter, glutamate (Henny et al. 2010). Orexins excite both dopaminergic and non-DA-ergic neurons of the VTA (Korotkova et al. 2003). Orexin-IR axon terminals are juxtaposed to dendrites and somata of dopamine neurons (Fadel and Deutch 2002) forming the A10 monoaminergic cell group in the mesencephalic tegmentum (Dahlstroem and Fuxe 1964). In addition, they influence GABA neurons of the VTA (Balcita-Pedicino and Sesack 2007). The orexin-IR boutons are rich in dense-core and electron lucent vesicles and establish *bona fide* synapses with VTA neurons of diverse phenotypes (Balcita-Pedicino and Sesack 2007). The relatively low number of detected synaptic specializations, however, has raised the possibility of regulatory orexin actions via volume transmission (Balcita-Pedicino and Sesack 2007). The mPFC receives a substantial DA input from the VTA via the mesocortical DA pathway (Dahlstroem and Fuxe 1964). DA terminals of VTA origin gate intrinsic inhibition in the mPFC and may enhance the firing of prelimbic neurons (Buchta et al. 2017). It is of note, the a subpopulation of VTA DA neurons is capable of co-releasing glutamate (Morales and Margolis 2017) in the PFC which targets fast-spiking GABAergic interneurons (Kabanova et al. 2015) as well as principal, pyramidal neurons (Pérez-López et al. 2018). The interconnection of these brain loci is reciprocal (Tzschentke and Schmidt 2000). Afferents from the mPFC synapse dominantly on projecting DA neurons of the VTA (Carr and Sesack 2000). In the prefrontal cortex, DA regulates cognition, learning, memory, motor events and reward, among others, mainly via D1 and D2 receptors (Tzschentke and Schmidt 2000; Tzschentke 2001).

Fluorescent retrograde labeling from the prefrontal cortex has revealed that the highest number of the projecting neurons were located in the rostral portion of the VTA, although the labeling extended nearly to its caudal end (Chandler et al. 2013). Both dopaminergic and chemically non-identified neurons accumulated the retrograde tracer. Our results also show that the vast majority of the projecting neurons belong to the dopamine phenotype (68.3%), while the rest of the cells presumably supplies the mPFC with GABA (Yokofujita et al. 2008) and glutamate (Gorelova et al. 2012). The simultaneous retrograde tract tracing from the mPFC (Fluoro-Gold injection) and the nucleus accumbens (Neuro-Dil injections) has shown that separate, projecting DA cell clusters are responsible for the innervation of these targets and the number of bidirectionally projecting DA neurons is negligible (Margolis et al. 2006). In our present viral labeling study, the retrogradely-traced DA neurons appeared in all subregions of the VTA, however, the prevalence of the retrogradely-traced cells varied in the different subdivisions. Most of the double labeled cells were in the IF, which represents the rostro-medial segment of the nuclear complex. Breton and co-workers have analyzed the origin of DA input from the VTA to the prelimbic and infralimbic cortices, the main constituents of the mPFC (Breton et al. 2019). The projections to both targets were predominantly ipsilateral, arising from midline regions of the VTA and almost 40% of the projecting cell were for them immunoreactive for TH. We have found that more than 60% of the projecting neurons were TH-positive. The difference might be due to the difference in the analyzed areas (selected ROIs in the current study vs. full VTA in the Breton’s study) or the tracing technique applied.

Orexin powerfully activates mPFC-projecting DA neurons—evidenced by increased c-Fos expression—in the caudo-medial part of VTA (Vittoz et al. 2008). Furthermore, its direct injection into the VTA selectively increased the dopamine efflux within the prefrontal cortex, the time spent grooming and spent awake (Vittoz and Berridge 2006). These data collectively suggest that orexin can regulate the dopamine neurotransmission in the mPFC via acting on DA neurons of the VTA. In another study, nicotine-activated DA neurons of the VTA were found infrequently contacted by orexin axons contrasting tyrosine hydroxylase-IR neurons of the locus coeruleus (Dehkordi et al. 2017). It is noteworthy, that non-dopaminergic neurons residing mainly in the parabrachial nucleus of the ipsilateral VTA also project to the mPFC (Del Cid-Pellitero and Garzón 2014).

Our current observations indicate that mPFC-projecting neurons of the VTA are intensely innervated by orexin-IR axons. Almost 70% of the projecting DA neurons received juxtaposed orexin-IR nerve fibers targeting both the dendrites and cell bodies of DA cells. The mPFC-projecting DA neurons innervated by the orexin system, represented about 5% of all orexin-innervated DA neuron population. Among the subregions of the VTA, the heaviest interneuronal communication was observed in the PBP and IF subunits.

The physiological role of orexin upon the modulation of PFC-projecting DA neurons is complex. The response of tegmental dopamine neurons to the activation of the PFC is controlled by orexin. Accordingly, the injection of orexin into VTA DA neurons increased baseline activity of the cells and augmented the excitatory responses to mPFC stimulation (Moorman and Aston-Jones 2010). Orexin input to the VTA is also crucial in the development of cocaine-induced plasticity of its dopamine neurons (Borgland et al. 2006) and for learning morphine-stimulus associations and preference (Aston-Jones et al. 2009, 2010).

### Dopaminergic neurons of the VTA targeting the medial division of shell of nucleus accumbens are innervated by orexinergic axons

The nucleus accumbens receives an intense dopaminergic innervation from the midbrain via the mesolimbic DA projection (Hasue and Shammah-Lagnado 2002; Björklund and Dunnett 2007). The substantia nigra (A9) and VTA (A10) both participate in the innervation of the nucleus. Dopamine neurons are also known to co-release glutamate (Dal Bo et al. 2004) and targeting of vGLUT2 in mature DA-ergic neurons has been reported to decrease the mesoaccumbal glutamatergic neurotransmission (Papathanou et al. 2018). The shell region of the nucleus accumbens receives DA and non-DA inputs form the VTA (Fallon and Moore 1978; Swanson 1982; Brog et al. 1993). In line with the previous reports, we also detected retrogradely labeled neurons in the VTA after targeting the medial part of the shell compartment by the Cav2-cre virus. Of all sampled dopaminergic neurons, 26% projected to the mNAcs and 79.6% of the projecting neurons were dopaminergic. The latter finding confirms an earlier report suggesting that the vast majority (80%) of the nucleus accumbens-projecting neurons are TH-immunoreactive (Hasue and Shammah-Lagnado 2002). Furthermore, we found that the main sources of DA neurons innervating the mNAcs are in the PBP, IF and PN nuclei of the VTA. A similar distribution of mNAcs-projecting, DA neurons has been reported using combined TH-immunocytochemistry and Fluoro-Gold retrograde labeling (Hasue and Shammah-Lagnado 2002). Dopamine neurotransmission in the nucleus accumbens has been implicated in the regulation of heroin reinforcement (Corre et al. 2018), rewarding and aversive behaviors (Danjo et al. 2014), obesity (Fulton et al. 2006), depression-related behaviors (Chaudhury et al. 2013) sexual preference (Beny-Shefer et al. 2017) and impulsive behavior (Flores-Dourojeanni et al. 2021), among others.

The dopaminergic VTA-nucleus accumbens neuronal axis is under the regulatory influence of orexin. Via targeting the NAc-projecting DA cells, orexin regulates reward seeking (Harris et al. 2005), cocaine self-administration (España et al. 2011), palatable food intake (Zheng et al. 2007), male sexual behavior (Muschamp et al. 2007), morphine withdrawal (Sharf et al. 2008) and controls the disinhibition of dopaminergic neurons in severe obesity (Tunisi et al. 2021).

In this study, we elucidated the structural background of this regulatory mechanism. Of all sampled orexin-innervated dopamine neurons of the VTA, 34.5% projected toward mNAcs. This innervation pattern is seven times higher than that of the mPFC. In the light of about equal numbers of orexin axons contacting mNAcs- and mPFC-projecting DA neurons and the several times higher percentage of DA neuron input to the mNAcs, orexin seems to exert a more intense drive and regulatory influence upon the nucleus accumbens than the mPFC. Of all counted, orexin-innervated DA neurons, a large percentage (64.4%) sends innervating axons to the mNAcs. This morphological finding indicates that in addition to the direct regulatory role of orexin (Trivedi et al. 1998; Lei et al. 2019; Li et al. 2021) on the neuronal circuits of the nucleus accumbens, the presented indirect orexin control via the VTA-to the nucleus accumbens is also significant. It is of note, that dopamine neurons of the human VTA have also been found intensely innervated by orexin-IR axons (Hrabovszky et al. 2013).

### Effects of orexin A on firing of dopaminergic neurons controlling the medial prefrontal cortex and the shell of nucleus accumbens

Both subsets of dopaminergic neurons were similarly driven to fire by exogenous orexin application. Notably, these effects were observed in the presence of blockers for AMPA, kainate and GABA-A receptors. This suggests that the effects of orexin onto these two VTA dopamine neuronal populations are likely mediated by a direct depolarizing action of orexin 1 and/or 2 receptors on the postsynaptic membranes, and not by ionotropic synaptic integration. In accordance, a previous study showed that orexin A, at the same concentration of 100 nM, increased firing of VTA dopamine neurons projecting to the NAcs in the presence of glutamatergic and GABAergic ionotropic receptor blockers (Baimel et al. 2017). This effect was blocked by an antagonist for the Orexin 1 receptor. Moreover, this study reported the absence of an effect of orexin A on the firing rate of VTA dopamine neurons projecting to the basolateral amygdala but did not test the effect of orexin A on VTA dopamine neurons projecting to the mPFC (Baimel et al. 2017). Our current finding elaborates on this and show that also VTA dopamine neurons innervating the mPFC are activated by orexin. This is also in accordance with a microdialysis study in awake rats showing that intracerebroventricular orexin A increased dopamine signaling in the mPFC (Vittoz and Berridge 2006). Our work thus provides insight in the underlying cellular mechanisms behind orexin control over dopamine release in medial prefrontal cortex and nucleus accumbens.

In summary, the present findings indicate that the hypothalamic orexin system innervates dopamine neurons of the VTA projecting both to the medial prefrontal cortex and the medial division of the shell part of the nucleus accumbens. The innervation density of DA neurons by orexin fibers is similar within the mesocortical and mesolimbic projections, and orexin equally excites both systems. Although, the exerted regulatory drive of the orexin system seems to be heavier upon the mesolimbic DA projection than via the mesocortical DA pathway, primarily due to the more intense wiring of DA cells with the nucleus accumbens.

## Data Availability

Data are available on reasonable request.

## Abbreviations

AAV: Adeno-associated virus
ACSF: Artificial cerebrospinal fluid
CAV-2: Canine adenovirus type 2
Cg1: Cingulate cortex, region 1
ChR-2: Channelrhodopsin-2
cp: Cerebral peduncle
CPu: Caudate-putamen
DA: Dopamine
fmi: Forceps minor
fr: Fasciculus retroflexus
HSV: Herpes simplex virus
IF: Interfascicular nucleus
IL: Infralimbic area
IPC: Interpeduncular nucleus, caudal subdivision
IPL: Interpeduncular nucleus, lateral subdivision
IPR: Interpeduncular nucleus, rostral subdivision
IR: Immunoreactive
LH: Lateral hypothalamus
ml: Medial lemniscus
mp: Mammillary peduncle
mPFC: Medial prefrontal cortex
MT: Medial terminal nucleus
NAc: Nucleus accumbens
mNAcs: Shell part of nucleus accumbens, medial division
PBP: Parabrachial pigmented subdivision of ventral tegmental area
PBS: Phosphate-buffered saline
PFA: Paraformaldehyde
PFC: Prefrontal cortex
PIF: Parainterfascicular nucleus
PN: Paranigral nucleus
PrL: Prelimbic area
Rli: Rostral linear nucleus
SNc: Substantia nigra, pars compacta
SNr: Substantia nigra, pars reticulata
TH: Tyrosine hydroxylase
